# Upregulation of miR-107 expression following hyperbaric oxygen treatment suppresses HMGB1/RAGE signaling in degenerated human nucleus pulposus cells

**DOI:** 10.1186/s13075-019-1830-1

**Published:** 2019-01-31

**Authors:** Chi-Chien Niu, Song-Shu Lin, Li-Jen Yuan, Meng-Ling Lu, Steve W. N. Ueng, Chuen-Yung Yang, Tsung-Ting Tsai, Po-Liang Lai

**Affiliations:** 1 Department of Orthopaedic Surgery, Chang Gung Memorial Hospital, No 5, Fu-Hsing Street 333, Taoyuan, Taoyuan, Taiwan; 2 Department of Orthopaedic Surgery, E-Da Hospital/I-Shou University, Kaohsiung, Taiwan; 3 College of Medicine, Chang Gung University, Taoyuan, Taiwan; 4 Department of Nursing, Chang Gung University of Science and Technology, Taoyuan, Taiwan; 5 Department of Orthopaedic Surgery, Chang Gung Memorial Hospital, Kaohsiung, Taiwan; 6 Bone and Joint Research Center, Chang Gung Memorial Hospital, Linkou, Taiwan

**Keywords:** Hyperbaric oxygen, Nucleus pulposus cells, miR-107, HMGB1, RAGE

## Abstract

**Background:**

The expression of both high-mobility group box 1 (HMGB1) and receptor for advanced glycation end-products (RAGE) is upregulated in degenerated discs. HMGB1 is known to function as a coupling factor between hypoxia and inflammation in arthritis, and this inflammatory response is modulated by microRNAs (miRNAs), with miR-107 expression downregulated during hypoxia. In this study, we investigated the regulation of the miR-107/HMGB1/RAGE pathway in degenerated nucleus pulposus cells (NPCs) after hyperbaric oxygen (HBO) treatment.

**Methods:**

NPCs were separated from human degenerated intervertebral disc tissues. The control cells were maintained in 5% CO_2_/95% air, and the hyperoxic cells were exposed to 100% O_2_ at 2.5 atmospheres absolute. MiRNA expression profiling was performed via microarray and confirmed by real-time PCR, and miRNA target genes were identified using bioinformatics and luciferase reporter assays. The cellular protein and mRNA levels of HMGB1, RAGE, and inducible nitric oxide synthase (iNOS) were assessed, and the phosphorylation of MAPK (p38MAPK, ERK, and JNK) was evaluated. Additionally, cytosolic and nuclear fractions of the IκBα and NF-κB p65 proteins were analyzed, and secreted HMGB1 and metalloprotease (MMP) levels in the conditioned media were quantified.

**Results:**

Using microarray analyses, 96 miRNAs were identified as upregulated and 66 downregulated following HBO treatment. Based on these results, miR-107 was selected for further investigation. Bioinformatics analyses indicated that the 3′ untranslated region of the HMGB1 mRNA contained the “seed-matched-sequence” for hsa-miR-107, which was validated via dual-luciferase reporter assays. MiR-107 was markedly induced by HBO, and simultaneous suppression of HMGB1 was observed in NPCs. Knockdown of miR-107 resulted in upregulation of HMGB1 expression in HBO-treated cells, and HBO treatment downregulated the mRNA and protein levels of HMGB1, RAGE, and iNOS and the secretion of HMGB1. In addition, HBO treatment upregulated the protein levels of cytosolic IκBα and decreased the nuclear translocation of NF-κB in NPCs. Moreover, HBO treatment downregulated the phosphorylation of p38MAPK, ERK, and JNK and significantly decreased the secretion of MMP-3, MMP-9, and MMP-13.

**Conclusions:**

HBO inhibits pathways related to HMGB1/RAGE signaling via upregulation of miR-107 expression in degenerated human NPCs.

## Introduction

The process of intervertebral disc degeneration (IDD) is believed to have a biochemical basis that involves inhibition of nuclear proteoglycan synthesis and enhanced matrix degradation caused by chemical mediators, such as interleukin (IL)-1, nitric oxide (NO), and matrix metalloproteinases (MMPs) [1, 2]. Increased NO levels inhibit proteoglycan synthesis in human lumbar disc cells [3] and play an essential role in the pathogenesis of disc degeneration by mediating the apoptosis of disc cells [4]. The increased expression and synthesis of MMPs and inflammatory factors is mediated by specific signal transduction pathways, including the nuclear factor-kappa B (NF-κB) and mitogen-activated protein kinase (MAPK)-mediated pathways [5]. Phosphorylation of MAPK results in the expression of inflammatory response factors, such as tumor necrosis factor-alpha (TNF-a) and NO [6, 7]. Blocking MAPK signaling decreases interleukin-1 (IL-1) and TNF-α induction of MMP-3, IL-6, and prostaglandin E2 (PGE2) in nucleus pulposus cells (NPCs) [8].

MicroRNAs (miRNAs) are endogenous non-coding small RNAs consisting of 20–25 nucleotides that serve to mediate gene regulatory events by pairing with the 3′ untranslated region (3′ UTR) of their target messenger RNAs (mRNAs) and, thus, modulating their expression. As such, miRNAs regulate diverse cellular processes, including cell proliferation, cell apoptosis, and cell differentiation [9]. Various miRNAs are deregulated in IDD and functionally implicated in its pathogenesis. MiR-21 promotes human NPC proliferation by affecting PTEN/AKT signaling [10]. MiR-10b promotes NP cell proliferation via the RhoC-Akt pathway by targeting HOXD10 during IDD [11], and miR-146a reduces IL-1–dependent inflammatory responses in intervertebral discs (IVDs) [12]. Additionally, miR-155 promotes Fas-mediated apoptosis by targeting Fas-associated protein with death domain (FADD) and caspase-3 during human IDD [13]. These findings demonstrate the direct involvement of miRNAs in the pathogenesis of degenerative disorders; however, the exact role of miR-107 in IVD has not been well defined.

High-mobility group box 1 (HMGB1), a ubiquitous non-histone DNA-binding protein, is an important modulator of inflammation [14]. Recently, HMGB1 has been shown to function as a potent pro-inflammatory mediator in degenerated human discs [15, 16]. Molecular analysis of cultured cells has demonstrated a significant increase in HMGB1 expression in highly degenerated discs, with herniated specimens showing greater HMGB1 expression levels than control specimens [15]. Moreover, HMGB1 promotes the release of inflammatory cytokines and the expression of MMPs in human IVD cells [16]. RAGE (receptor for advanced glycation end-products) is upregulated under inflammatory conditions in IVD cells in vitro [17] and in vivo [18], and extracellular HMGB1 interacts with RAGE present on the membrane of nearby cells to activate NF-κB or MAPKs, which are key factors in the inflammatory response [5, 19].

Human IVD is the largest avascular tissue in the body, and it receives all essential nutrients, such as oxygen and glucose, through the cartilage endplate [20]. During aging and degeneration of the IVD, especially calcification of the end plate, the flow of nutrients and metabolites is reduced [21]. The oxygen tensions may show a further decreased in degenerated IVD than healthy IVD.

Hypoxia induces MAPK activity in rat NPCs [22], and hypoxia is also a potent inducer of extracellular HMGB1 and may, in turn, play an important role in the development of arthritis [23]. Depending on the cell type, miR-107 expression is either downregulated [24], increased, or unchanged in the presence of hypoxia [25] or increased under conditions of hyperoxia [26]. Additionally, miR-107 expression has been shown to be downregulated in cartilage from patients with osteoarthritis (OA) [27, 28].

Hyperbaric oxygen (HBO) treatment can serve to improve hypoxic conditions by increasing tissue and/or microvascular O_2_ levels [6]. In addition, HBO treatment increased the O_2_ dissolved in cell culture medium. In this study, we demonstrated that HBO treatment increased miR-107 expression in degenerated NPCs, as assessed via microarray analysis and confirmed by real-time PCR. We used bioinformatics to identify putative target sequences for miR-107 in the human HMGB1 mRNA and confirmed these by way of luciferase reporter assays. Finally, we investigated the effects of HBO treatment on the miR-107/HMGB1/RAGE signaling-mediated catabolic pathway in degenerated human NPCs.

## Materials and methods

The experimental protocol was approved by the Human Subjects Institutional Review Board at Chang Gung Memorial Hospital, Taiwan.

### Cell isolation and cultivation

Fresh abnormal disc tissues were harvested from the degenerated lumbar IVDs of patients who underwent total discectomy and posterior lumbar interbody fusion (Table 1). NPCs were separated from the nucleus tissue by performing sequential enzymatic digestion, first with 0.4% pronase (Sigma, USA) for 1 h and then with 0.025% collagenase P (Boehringe, Germany) and 0.004% DNase II (Sigma, USA) at 37 °C overnight. After digestion, the cells were washed with DMEM/F-12 and seeded in three fresh T-75 flasks at a density of 5000 cells/cm^2^ and incubated in a humidified atmosphere of 5% CO_2_ and 95% air until the cells attained confluence.

**Table 1:** Patient demographics

Patient	Sex	Age	Degree of degeneration	Experiment
1	F	58	Pfirrmann grading 4	MiRNA profiling
2	F	67	Pfirrmann grading 4	Real-time PCR
3	F	46	Pfirrmann grading 5	Reporter assay
4	F	41	Pfirrmann grading 4	Western blot
5	M	61	Pfirrmann grading 5	MiRNA profiling
6	F	72	Pfirrmann grading 5	Real-time PCR
7	F	74	Pfirrmann grading 4	Reporter assay
8	F	70	Pfirrmann grading 4	MiRNA profiling
9	F	66	Pfirrmann grading 4	ELISA
10	F	56	Pfirrmann grading 4	Western blot
11	M	76	Pfirrmann grading 5	MiRNA profiling
12	F	48	Pfirrmann grading 4	ELISA
13	M	75	Pfirrmann grading 5	Real-time PCR
14	M	69	Pfirrmann grading 5	Real-time PCR
15	M	77	Pfirrmann grading 4	Reporter assay
16	M	79	Pfirrmann grading 5	Western blot
17	F	75	Pfirrmann grading 5	Western blot
18	F	68	Pfirrmann grading 4	ELISA
19	M	61	Pfirrmann grading 4	Real-time PCR
20	M	77	Pfirrmann grading 5	ELISA
21	F	69	Pfirrmann grading 5	ELISA
22	F	58	Pfirrmann grading 4	Real-time PCR
23	F	69	Pfirrmann grading 5	MAPK
24	F	65	Pfirrmann grading 5	Western blot
25	F	63	Pfirrmann grading 4	Real-time PCR
26	F	82	Pfirrmann grading 5	ELISA
27	M	57	Pfirrmann grading 4	MAPK
28	F	67	Pfirrmann grading 5	Real-time PCR
29	M	35	Pfirrmann grading 4	Western blot
30	F	66	Pfirrmann grading 4	Western blot
31	M	67	Pfirrmann grading 4	Western blot
32	F	64	Pfirrmann grading 5	Western blot
33	F	77	Pfirrmann grading 5	ELISA
34	F	41	Pfirrmann grading 4	Reporter assay
35	F	51	Pfirrmann grading 4	MAPK
36	M	78	Pfirrmann grading 5	Real-time PCR
37	F	68	Pfirrmann grading 4	Real-time PCR
38	F	45	Pfirrmann grading 4	ELISA
39	F	59	Pfirrmann grading 4	Real-time PCR
40	M	61	Pfirrmann grading 4	MAPK
41	F	64	Pfirrmann grading 5	Western blot
42	F	78	Pfirrmann grading 5	Real-time PCR

Cells were used at passage 2 for each experiment.

### Cells exposure to intermittent HBO

Approximately 2 × 10^5^ cells were plated on a 100-mm cell culture dish containing 10 ml DMEM/F-12 supplemented with 10% FBS. The cultures were maintained at 37 °C in a humidified atmosphere of 5% CO_2_ and 95% air. The cells were either maintained in 5% CO2/95% air throughout the experiment as a control or in HBO-treated protocol containing three times of 100% O_2_ at 2.5 ATA (atmospheres absolute) for 25 min each, two times of air break (5% CO2/95% air) at 2.5 ATA for 5 min each, and transfer times in a hyperbaric chamber (Sigma II; Perry Baromedical, USA). The total duration for HBO-treated protocol is about 120 min. HBO treatment administered a total of 120 mins every 48 h. At 24 h after each treatment, 10 ml of the conditioned media (CM) was collected and stored at − 70 **°C** for further analysis.

### MiRNA profiling

After HBO treatment, total RNA was isolated using a mirVana miRNA isolation kit (Ambion, USA). MiRNA expression profiling was accomplished using TaqMan Human MicroRNA Array A and B Cards containing 754 mature human microRNAs (Applied Biosystems, USA) and an ABI 7900 real-time PCR System according to the manufacturer’s protocol. MiRNA expression profiling was performed on eight samples from four patients (with or without HBO treatment). Briefly, 3 μL of total RNA from each sample was reverse-transcribed using the TaqMan miRNA Reverse Transcription Kit (Applied Biosystems, USA) and the stem-loop Megaplex Primer Pool Sets (set A or B). A total of 7.5 μL of reaction mixture was immediately incubated under the following conditions: 40 cycles at 16 °C for 2 min, 42 °C for 1 min, and 50 °C for 1 s, and 85 °C for 5 min. Then, 2.5 μL of the resultant Megaplex RT products were mixed with 2.5 μL of Megaplex PreAmp Primers (pool A or B) and 12.5 μL of TaqMan PreAmp Master Mix. A total of 25 μL of the reaction mixture was incubated using the following program: 95 °C for 10 min, 55 °C for 2 min, and 72 °C for 2 min followed by 12 cycles at 95 °C for 15 s, 60 °C for 4 min, and 99.9 °C for 10 min. The pre-amplified cDNA was diluted with 0.1× TE (10 mM Tris, pH 8.0, 1 mM EDTA) to 100 μL and used for PCR.

### Real-time PCR

TaqMan miRNA assays (ABI PRISM; Applied Biosystems, USA) were used to detect the expression levels of the mature miR-107. For the reverse transcription (RT) reactions, 10 ng of total RNA was mixed with the RT primer. RT reactions were performed at 16 °C for 30 min, 42 °C for 30 min, 85 °C for 5 min, and then maintained at 4 °C. Following the RT reactions, 1.5 μl of cDNA was used for a polymerase chain reaction (PCR) using 2 μl of TaqMan primers. The PCR was conducted at 95 °C for 10 min followed by 40 cycles of 95 °C for 15 s and 60 °C for 60 s in an ABI 7900 real-time PCR system (Applied Biosystems, USA). The quantitative PCR results were analyzed and expressed as the relative miRNA level using the U6 snRNA for normalization purposes. The fold change in the miRNA expression in each sample relative to the average expression in the control was calculated based on the threshold cycle (CT) value using the following formula: relative gene expression = 2^−ΔΔCt^, where − ΔΔCt = (Ct gene of interest − Ct internal control gene) treated − (Ct gene of interest − Ct internal control gene) untreated. The experiment was performed four times, and the mean relative fold change ± standard deviation was determined.

### MiRNA target prediction and dual-luciferase reporter assays

Target Scan 7.1 (http://www.targetscan.org) and miRnalyze (http://www.mirnalyze.in) online software were used to analyze the putative target genes of miR-107. The 3′ UTR of HMGB1 containing the miR-107 binding site was cloned into pmirGLO dual-luciferase miRNA reporter vectors (Promega, USA). A mutated 3′ UTR of HMGB1 was introduced into the potential miR-107 binding site. The reporter vectors containing the wild-type or mutant HMGB1 3′ UTR were transfected into NPCs using Lipofectamine 2000 (Invitrogen, USA). After incubation with or without HBO, transfected cells were lysed. Firefly and Renilla luciferase activities were detected using the dual-luciferase assay system (Promega, USA) in accordance with the manufacturer’s instructions.

### Transfection of NPCs with anti-miRNAs and analysis after HBO treatment

NPCs were seeded into six-well plates at a density of 2 × 10^5^ cells/well in culture medium without antibiotics. The next day (day 1), cells were transfected with anti-miR-107 (100 nM; Ambion, USA) using RNAiMAX (Invitrogen, USA) and cultured in an incubator at 37 °C with 5% CO2. After 8 h of transfection, the culture medium was changed to DMEM/F-12 with 10% FBS, and the cells were exposed to HBO treatment. On days 3 and 5, the cells were re-transfected once and exposed to HBO.

At 12 h after the third HBO treatment, cellular RNA was isolated using an RNeasy mini kit (Qiagen, USA) and reverse-transcribed into cDNA with the ImProm-II reverse transcription system (Promega, USA). For real-time PCR detection of HMGB1 transcripts, cDNA was analyzed on an ABI PRISM 7900 sequence detection system using TaqMan PCR Master Mix (Applied Biosystems, USA). The cycle threshold (Ct) values were obtained, and the data were normalized to β-actin expression using the ΔΔCt method to calculate the relative mRNA levels of each target gene.

At 24 h after the third HBO treatment, cells were washed with PBS and extracted using M-PER mammalian protein extraction reagent (Thermo Fisher Scientific, USA). For immunoblotting experiments, the proteins were separated via SDS-PAGE and transferred onto nitrocellulose membranes using a protein transfer unit (Bio-Rad, USA). After blocking with 10% nonfat milk, the membranes were incubated overnight at 4 **°C** with a 1000-fold dilution of mouse antibodies against HMGB1 (Abcam, UK) and β-actin (Abcam, UK). After washing, the membranes were further incubated for 2 h with a 10,000-fold dilution of goat anti-mouse IgG conjugated to horseradish peroxidase (CalBiochem, USA). The membranes were then washed and rinsed with ECL detection reagents (Amersham Pharmacia Biotech, UK). The band images were photographed using Hyperfilm (Amersham Pharmacia Biotech, UK). The intensity of the staining for HMGB1 and β-actin was quantified using an image analysis system (Image-Pro plus 5.0; Media Cybernetics, USA).

### RNA extraction and real-time PCR detection of HMGB1, RAGE, and iNOS

NPCs were plated at a density of 3 × 10^5^ cells per 100-mm culture dish in 10 ml of DMEM/F-12 containing 5% FBS. At 12 h after the third HBO treatment, RNA extraction was performed as previously described for real-time PCR detection of the HMGB1, RAGE, and nitric oxide synthase (iNOS) mRNA transcripts**.**

### Protein extraction and Western blot analysis of HMGB1, RAGE, and iNOS

NPCs were plated at a density of 3 × 10^5^ cells per 100-mm culture dish in 10 ml of DMEM/F-12 containing 5% FBS. At 24 h after the third HBO treatment, cells were washed with PBS and extracted using M-PER mammalian protein extraction reagent (Thermo Fisher Scientific, USA). Protein extraction, separation, and transfer were performed as described above. After blocking with 10% nonfat milk, the nitrocellulose membranes were incubated overnight at 4 **°C** with a 1000-fold dilution of mouse antibody against HMGB1 (Abcam, UK), RAGE (Abcam, UK), iNOS (BD Biosciences, USA), or β-actin (Abcam, UK). After washing, the membranes were further incubated for 2 h with a 10,000-fold dilution of goat anti-mouse IgG conjugated to horseradish peroxidase (CalBiochem, USA). The membranes were then washed and rinsed with ECL detection reagents (Amersham Pharmacia Biotech, UK). The band images were photographed using Hyperfilm (Amersham Pharmacia Biotech, UK). The intensity of the staining for HMGB1, RAGE, and iNOS was quantified using an image analysis system (Image-Pro plus 5.0; Media Cybernetics, USA).

### HMGB1 ELISA assays

The post-treatment levels of HMGB1 in the CM were determined using an ELISA (Human HMGB1 ELISA Kit; LifeSpan BioSciences, USA). At 24 h after each treatment, 200 μl of CM was sampled and analyzed for HMGB1 levels according to the manufacturer’s instructions.

### MAPK phosphorylation assays

Phosphorylation levels of ERK, JNK, and p38 MAPK were measured following HBO treatment. At 15 and 30 min after the third HBO treatment, the cells were washed with PBS and trypsinzed. After mild centrifugation, the cell pellet was suspended in lysis buffer using the protocol and reagents from a Human Phosphor-Kinase Array Kit (R&D Systems, USA). Dot images were obtained using ECL Hyper film, and staining intensities were quantified using an image analysis system.

### Preparation of cytosolic and nuclear fractions for IκBα and NF-κB p65 detection

At 60 min after the third HBO treatment, the cells were rinsed with PBS, treated with 0.05% trypsin, and then collected by centrifugation. NE-PER nuclear and cytoplasmic extraction reagents (Thermo Fisher Scientific, USA) were used to isolate cytoplasmic and nuclear extracts from the cells. The proteins were separated by 10% SDS-PAGE. After blocking with 10% nonfat milk, the membranes were incubated overnight at 4 °C with a 1000-fold dilution of a mouse antibody against IκBα (Cell Signal, USA) or β-actin (Abcam, UK) for cytoplasmic extracts and NF-κB p65 (Cell Signal, USA) or the TATA binding protein (TBP; Abcam, UK) for nuclear extracts. The membranes were then washed and rinsed with ECL detection reagents. The band images were photographed using Hyperfilm. The intensity of the staining for IκBα, β-actin, NF-κB p65, and TATA was quantified using an image analysis system.

### MMP-3, MMP-9, and MMP-13 ELISA assays

The levels of MMP-3, MMP-9, and MMP-13 in the CM after hyperbaric or normbaric treatments were determined using an ELISA kit (Quantikine Human MMP-3, MMP-9, and MMP-13; R&D Systems, USA). At 24 h after each treatment, 200 μl of CM was sampled and analyzed for MMPs levels according to the manufacturer’s instructions. The results were normalized to 10^6^ cells.

### Statistical analysis

Data are expressed as the mean ± standard deviation (SD). The *p* value for the Student’s *t* test was calculated, and a *p* value of < 0.05 was considered statistically significant.

## Results

### Heat maps of miRNA expression in degenerated NPCs after HBO treatment

**Fig. 1:**
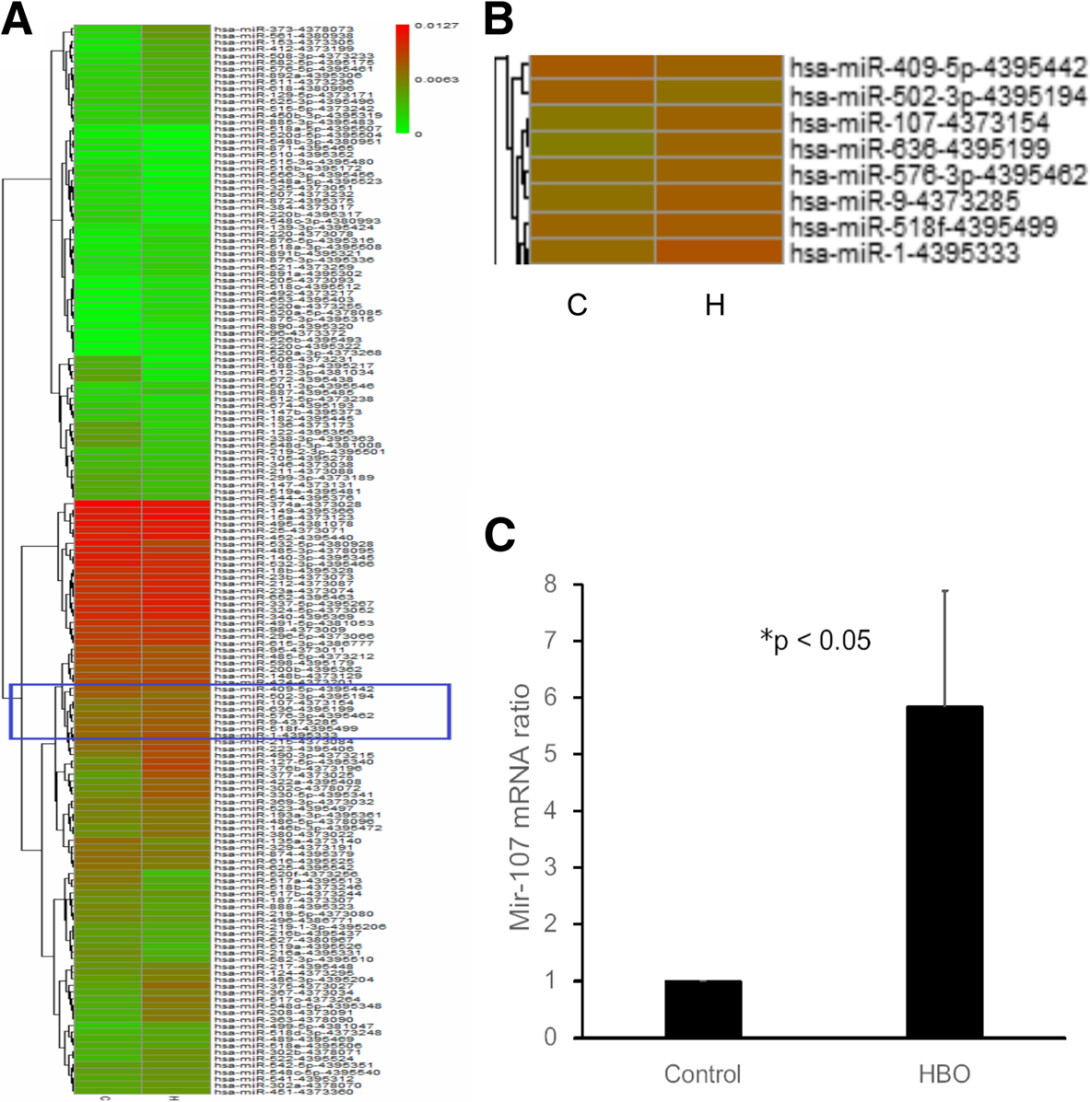
HBO modulates the expression of miRNAs in degenerated NPCs. **a** A heat map of the miRNAs with significantly changed expression levels in the HBO-treated group compared with the control group. Average expression is shown for each miRNA in each class. There were 96 miRNAs upregulated and 66 downregulated by at least 1.5-fold following HBO treatment. **b** MiR-107 was chosen for further investigation. **c** HBO treatment increased miR-107 expression in NPCs (*p < 0.05)

To identify the miRNAs involved in the molecular regulation of NPCs after HBO treatment, the miRNA expression profile of NPCs was performed using a TaqMan Human MiRNA Array A Card. As shown in Fig. 1a, there were 96 miRNAs upregulated and 66 downregulated by at least 1.5-fold following HBO treatment (*n* = 4). Among these (Fig. 1b), miR-107 was chosen for further investigation as previous studies revealed that miR-107 mediates an inflammation response [27] and its expression is lower in OA cartilage than in normal cartilage [25, 26].

### HBO treatment increased miR-107 expression in NPCs

HBO treatment increased miR-107 expression in NPCs (5.84 ± 2.04 fold, *p < 0.05, *n* = 4; Fig. 1c). These results indicated that miR-107 might play an important role in inhibiting the progression of IDD in NPCs after HBO treatment.

### Seed sequence of miR-107 in the 3’ UTR of the HMGB1 mRNA

**Fig. 2:**
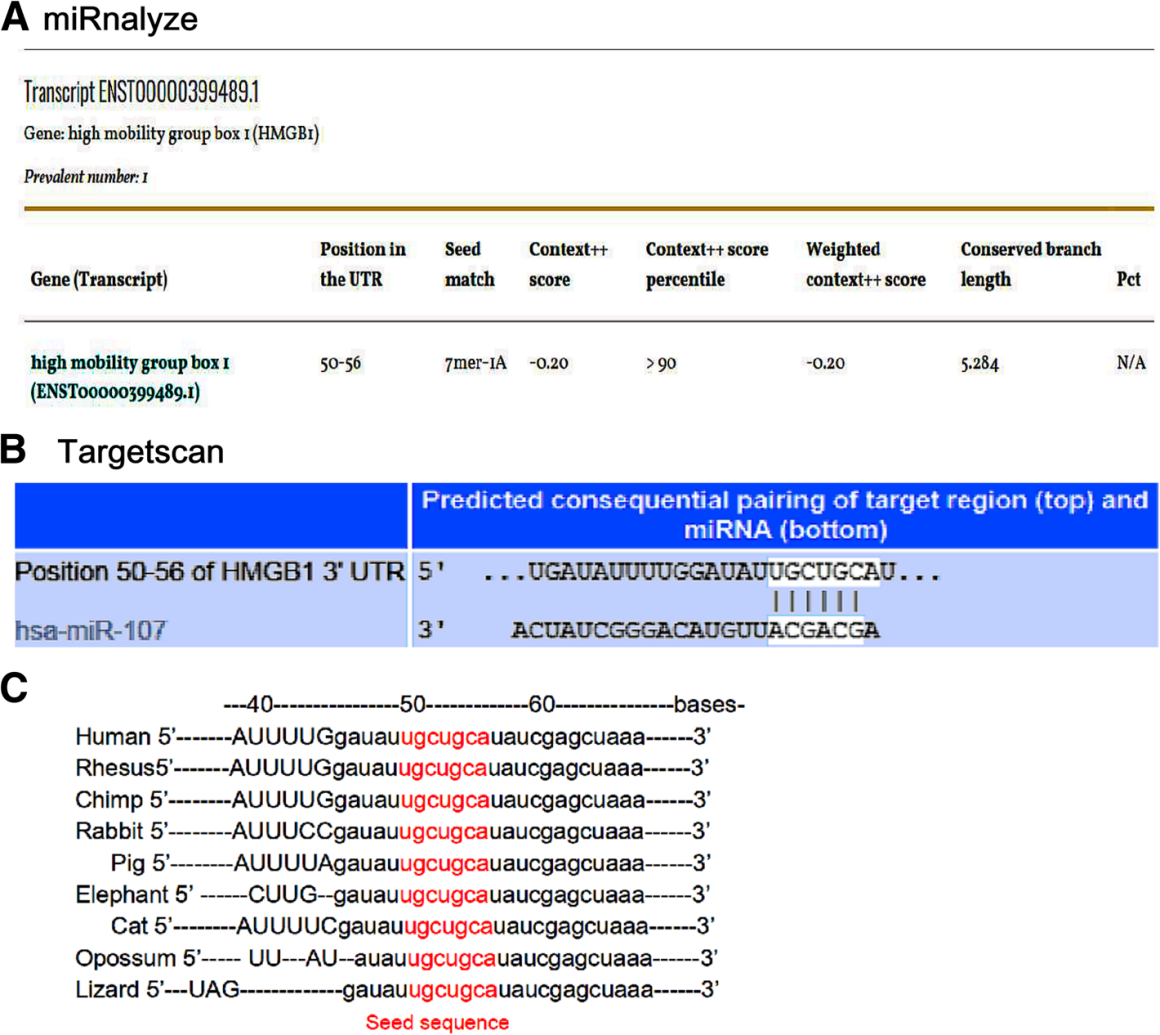
Seed sequence of miR-107 in the 3′ UTR of the HMGB1 mRNA. **a** MiRnalyze predicted the duplex of miR-107 with its seed sequence in the 3′ UTR of the human HMGB1 mRNA. **b** TargetScan predicted the duplex of miR-107 with the seed sequence in the 3′ UTR of the human HMGB1 mRNA. The sequences in white are the locations of the potential seed-matched sequences for the miRNAs assessed. **c** Cross-species conservation of the miR-107 seed sequence in the 3′ UTR of the human HMGB1 mRNA as identified via the TargetScan algorithm (sequences in red)

To investigate the potential molecular targets of miR-107, we screened for putative target genes of miR-107 using Target Scan 7.1 (http://www.targetscan.org) and miRnalyze (http://www.mirnalyze.in) online software. We found that HMGB1, an important regulator of inflammation, was likely a direct target of miR-107, as the 3′ UTR of HMGB1 contained a potential binding element for miR-107 with a 7-nt match to the miR-107 seed region (Fig. 2a,b). Additionally, cross-species conservation of the miR-107 seed sequence in the 3′ UTR of the HMGB1 mRNA was confirmed by the Target Scan algorithm (Fig. 2c). These findings suggested that hsa-miR-107 might target the HMGB1 mRNA by directly recognizing its seed-matched sequence present in the 3’ UTR.

### HMGB1 is a direct target of miR-107

**Fig. 3:**
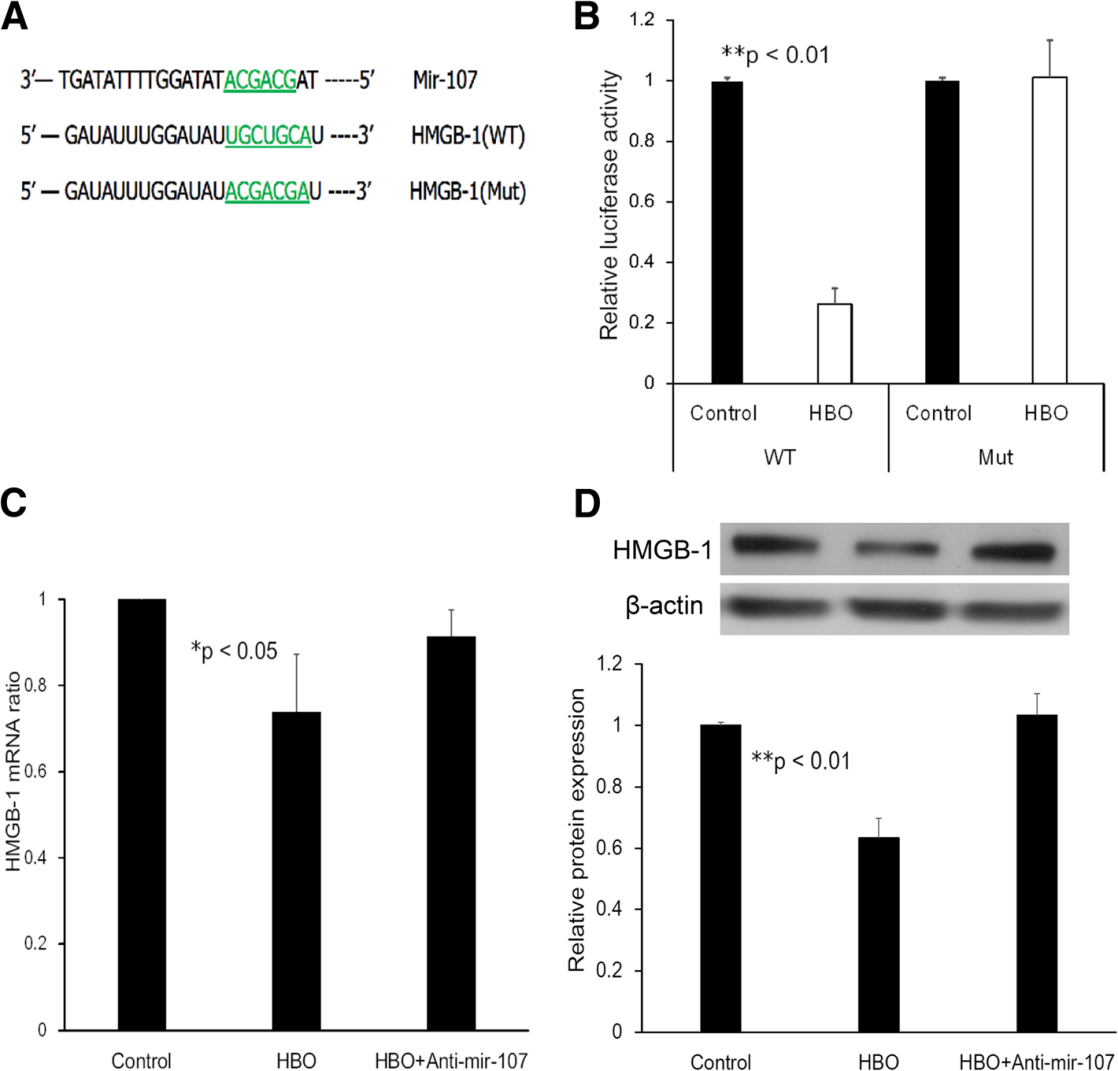
HMGB1 is a direct target of miR-107. **a** Diagram of the binding site between miR-107 and the HMGB1 3′ UTR. The reporter vectors contain the wild-type (WT) or mutant (Mut) HMGB1 3′ UTR. **b** Dual-luciferase reporter assay of the HMGB1 3′ UTR. The reporter vectors containing the WT or Mut HMGB1 3′ UTR were transfected into NPCs. Luciferase activity was shown to be significantly downregulated after HBO treatment (**p < 0.01; *n* = 4) in the constructs harboring the WT but not in the Mut 3′ UTR (*p* > 0.05, *n* = 4). **c** Real-time PCR analysis of HMGB1 mRNA expression in degenerated NPCs transfected with miR-107 inhibitors. HMGB1 mRNA expression was downregulated after HBO treatment (*p < 0.05; *n* = 4). Anti-miR-107 reversed the suppressive effects of HBO (*p* > 0.05; *n* = 4). **d** Western blot analysis of HMGB1 protein expression in NPCs transfected with miR-107 inhibitors. Values were normalized against β-actin. HMGB1 protein expression was significantly downregulated after HBO treatment (**p < 0.01; *n* = 4). MiR-107 inhibitors reversed the suppressive effects of HBO (*p* > 0.05; *n* = 4). *WT* wild type, *Mut* mutant

To validate the direct targeting of HMGB1 by miR-107, the wild type (WT) or a mutant variant (Mut) of the HMGB1 3′ UTR containing the target sequence was cloned into a dual-luciferase reporter vector (Fig. 3a). Overexpression of miR-107 after HBO treatment significantly inhibited luciferase activity of the WT HMGB1 3′ UTR (Fig. 3b, 0.26 ± 0.05 fold, **p < 0.01, *n* = 4), whereas mutation of the miR-107 binding sites abolished this inhibitory effect of miR-107 in the degenerated human NPCs (Fig. 3b, 1.01 ± 0.12 fold, *p* > 0.05, *n* = 4). These observations support the conclusion that HMGB1 is a target gene of miR-107 following HBO treatment.

We next examined the expression of HMGB1 in NPCs transfected with an anti-miR-107 construct. As shown in Fig. 3c, HBO treatment decreased the mRNA expression of HMGB1 (0.73 ± 0.12 fold, *p < 0.05, *n* = 4), whereas transfection with anti-miR-107 increased the mRNA level of HMGB1 in NPCs after HBO treatment (0.92 ± 0.06 fold, *p* > 0.05, *n* = 4). Western blot analysis was performed to examine the protein level of HMGB1 (Fig. 3d), and the results indicated that HBO treatment led to a significant decrease in the protein level of HMGB1 (0.63 ± 0.06 fold, **p < 0.01, *n* = 4), whereas knockdown of miR-107 upregulated HMGB1 protein expression in NPCs following HBO treatment (1.03 ± 0.07 fold, *p* > 0.05, *n* = 4). These data indicated that HMGB1 was negatively mediated by miR-107 at the post-transcriptional level in NPCs after HBO treatment, as overexpression of miR-107 after HBO treatment significantly inhibited the mRNA (Fig. 3c) and protein (Fig. 3d) expression of HMGB1 in these cells.

### Effect of HBO treatment on the mRNA and protein expression of HMGB1, RAGE, and iNOS

**Fig. 4:**
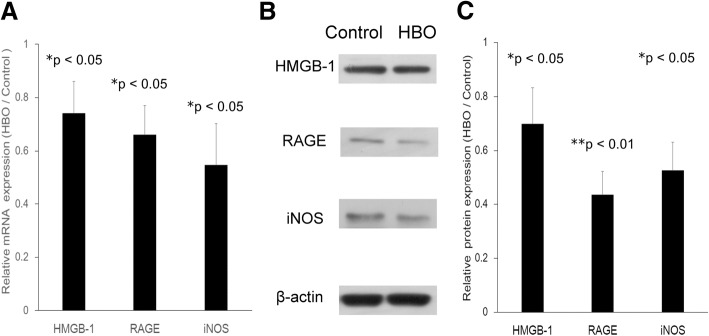
Effect of HBO on the mRNA and protein expression of HMGB1, RAGE, and iNOS. **a** HBO treatment significantly suppressed the mRNA expression of HMGB1 (*p < 0.05; *n* = 4), RAGE (*p < 0.05; *n* = 4), and iNOS (*p < 0.05; *n* = 4) in degenerated NPCs. **b** Western blot analysis was performed to examine the protein expression of HMGB1, RAGE, and iNOS. The protein expression of HMGB1 (*p < 0.05; *n* = 3), RAGE (*p < 0.01; *n* = 3), and iNOS (*p < 0.05; *n* = 3) were significantly downregulated after culturing for three rounds of HBO treatment. **c** The quantification of relative protein expression levels

Figure 4a shows that HBO treatment significantly suppressed the mRNA expression of HMGB1 (0.74 ± 0.12 fold, *p < 0.05, *n* = 4), RAGE (0.66 ± 0.11 fold, *p < 0.05, *n* = 4), and iNOS (0.54 ± 0.15 fold, *p < 0.05, *n* = 4) in NPCs, and the results of Western blot analysis presented in Fig. 4b show that the protein levels of HMGB1 (0.69 ± 0.13 fold, *p < 0.05, *n* = 3), RAGE (0.43 ± 0.08 fold, **p < 0.01, *n* = 3), and iNOS (0.52 ± 0.10 fold, *p < 0.05, *n* = 3) were downregulated after culturing for three rounds of HBO treatment. The quantification of the relative protein expression levels is depicted in Fig. 4c.

### Effect of HBO on the secretion of HMGB1

**Fig. 5:**
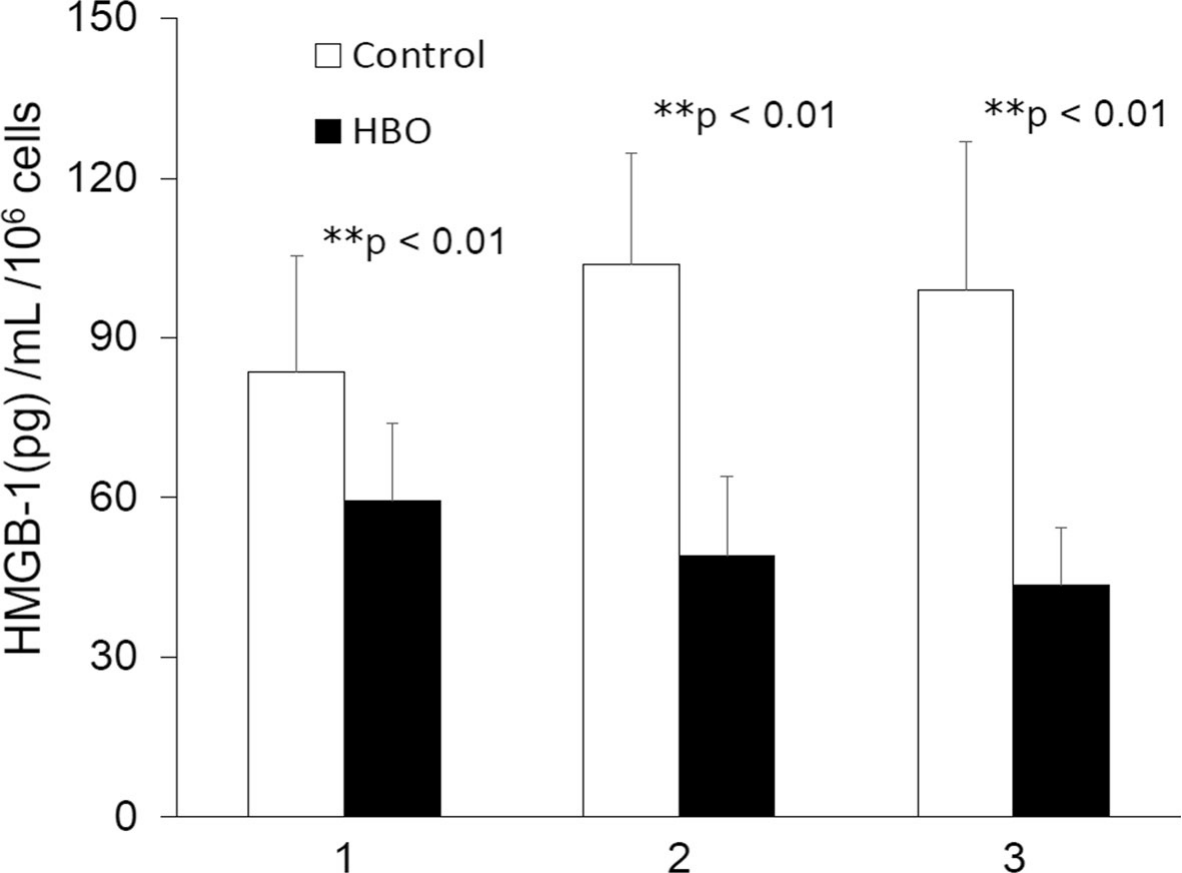
Effect of HBO on the secretion of HMGB1. HBO intervention significantly inhibited the extracellular release of HMGB1 by degenerated NPCs (**p < 0.01 in first, second, and third treatment)

Subsequently, we examined the effect of HBO on the extracellular release of HMGB1 by NPCs following each of three rounds of HBO treatment (Fig. 5, data are presented as mean ± SD, pg/mL; control group vs. HBO group: 83.6 ± 21.9 vs. 59.5 ± 14.4, **p < 0.01, *n* = 4; 103.7 ± 21.1 vs. 49.0 ± 15.0, **p < 0.01, *n* = 4; 99.0 ± 27.8 vs. 43.6 ± 10.8, **p < 0.01, *n* = 4; respectively). In accordance with the observed protein levels, it was apparent that HBO treatment significantly inhibited the extracellular release of HMGB1 by NPCs.

### Effect of HBO on MAPK phosphorylation

**Fig. 6:**
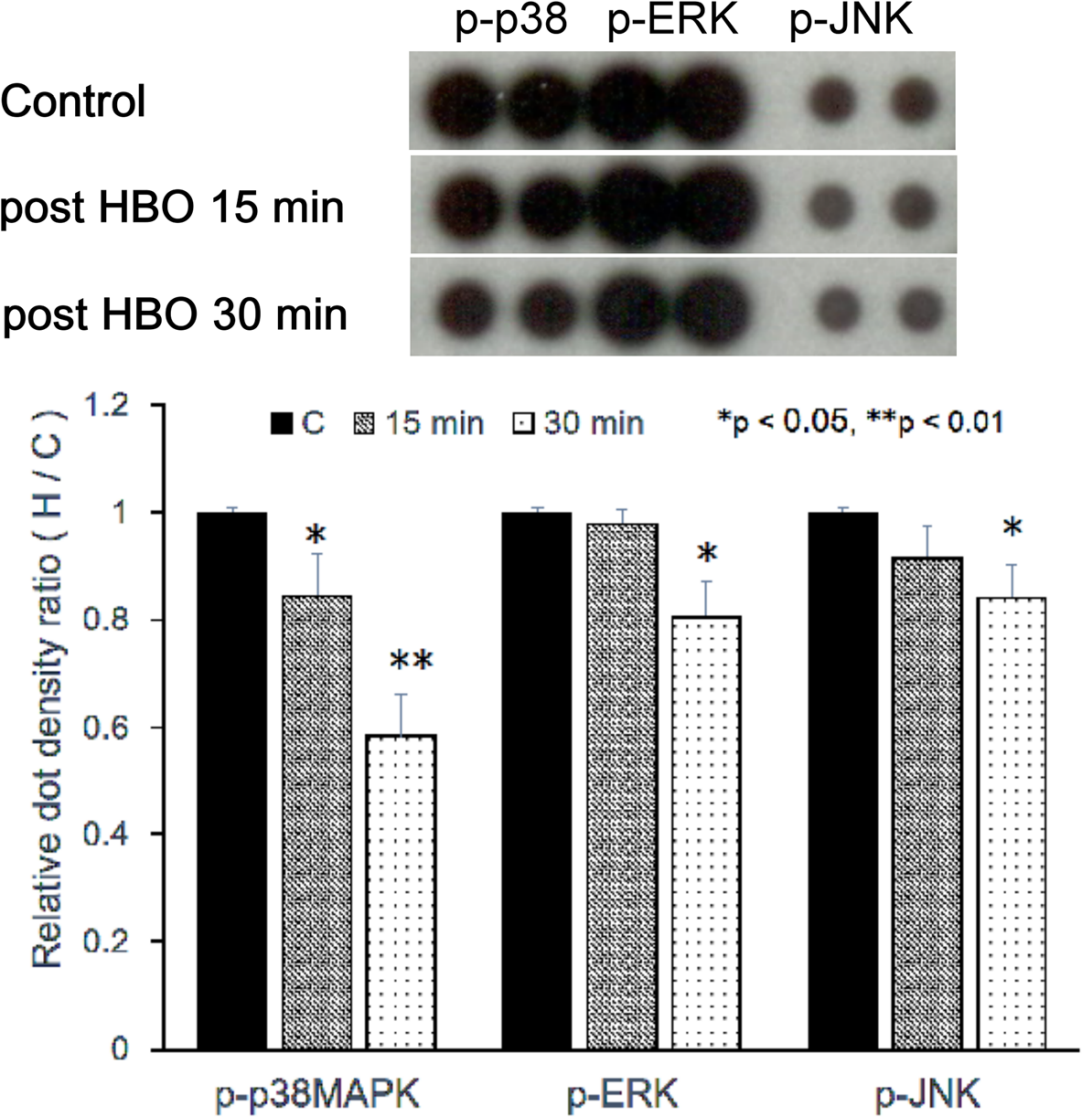
Effect of HBO on MAPK phosphorylation. HBO treatment significantly suppressed ERK, JNK, and p38 MAPK phosphorylation in degenerated NPCs. Phosphorylation dot density ratios for the HBO and control groups were determined at 15 and 30 min after the third round of HBO treatment. At 15 min, the ratio for p38 MAPK (*p < 0.05; *n* = 4) was significantly decreased. At 30 min, the ratio for p38 MAPK (**p < 0.01, *n* = 4), ERK (*p < 0.05, *n* = 4), and JNK (*p < 0.05, *n* = 4) were significantly decreased. These results indicated that HBO treatment significantly suppressed MAPK phosphorylation in degenerated NPCs

To assess the underlying molecular mechanisms affecting the catabolic pathways mediated by HMGB1/RAGE in NPCs after HBO treatment, we evaluated the effects of HBO on MAPK activity (Fig. 6). We counted the dot density ratios for the HBO and control groups at 15 and 30 min after administering the third HBO treatment. At 15 min, the ratio was 84.4% ± 7.9% for p38 MAPK phosphorylation (*p < 0.05, *n* = 4), 97.8% ± 2.5% for ERK phosphorylation (*p* > 0.05, *n* = 4), and 91.4% ± 6.1% for JNK phosphorylation (*p* > 0.05, *n* = 4). At 30 min, the ratio was 58.3% ± 7.7% for p38 MAPK phosphorylation (**p < 0.01, *n* = 4), 80.4% ± 6.9% for ERK phosphorylation (*p < 0.05, *n* = 4), and 84.0% ± 6.4% for JNK phosphorylation (*p < 0.05, *n* = 4). These observations indicated that HBO treatment significantly suppressed MAPK phosphorylation in NPCs.

### Effect of HBO on the protein expression of IκBα and NF-κB p65

**Fig. 7:**
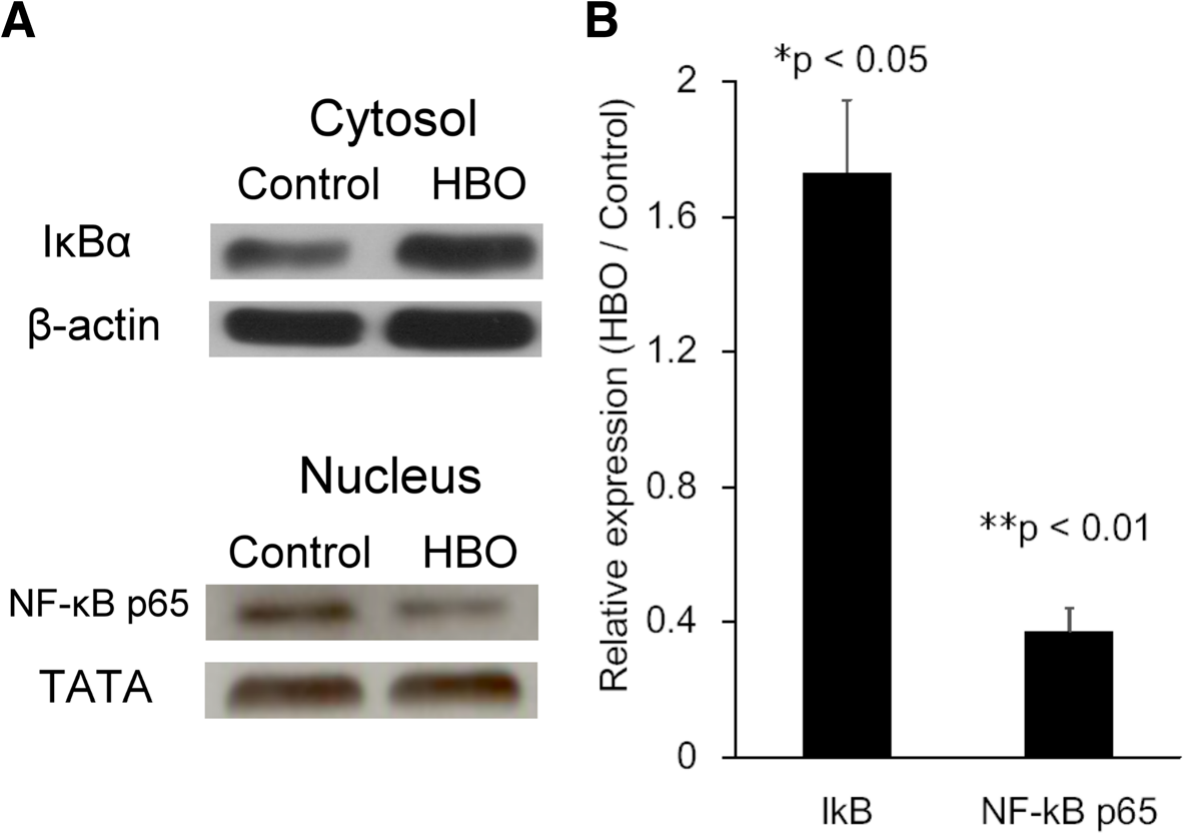
Effect of HBO on the protein expression of IκBα and NF-κB p65. **a** The cytosolic protein levels of IκBα were significantly increased at 60 min after the administration of the third HBO treatment (*p < 0.05, *n* = 3). In addition, the levels of NF-κB p65 in the nucleus were significantly decreased after HBO treatment (*p < 0.05, *n* = 3). IκBα resides in the cytoplasm to prevent NF-κB translocation to the nucleus, and HBO treatment appeared to decrease NF-kB inflammatory signaling. **b** Quantification of the relative protein expression levels

HMGB1 has been reported to function as an inducer of the NF-kB inflammatory signaling pathway. In this study, HBO treatment was shown to significantly suppress the mRNA expression of iNOS (Fig. 4a). As NF-κB is a central transcription factor that serves to regulate the expression of iNOS, the effects of HBO treatment on the nuclear translocation of the NF-κB p65 subunits were examined (Fig. 7). The protein expression of IκBα was up-regulated after HBO treatment (1.63 ± 0.18 fold, *p < 0.05, *n* = 3). In addition, the levels of NF-κB p65 in the nucleus (0.41 ± 0.06 fold, *p < 0.05, *n* = 3) was markedly decreased at 60 min after administration of the third HBO treatment (Fig. 7a). Figure 7b shows the quantification of the relative protein expression levels. NF-κB–mediated iNOS mRNA (Fig. 4a) and protein (Fig. 4b) expressions were markedly downregulated following HBO intervention.

### Effect of HBO on MMP-3, MMP-9, and MMP-13 secretion

**Fig. 8:**
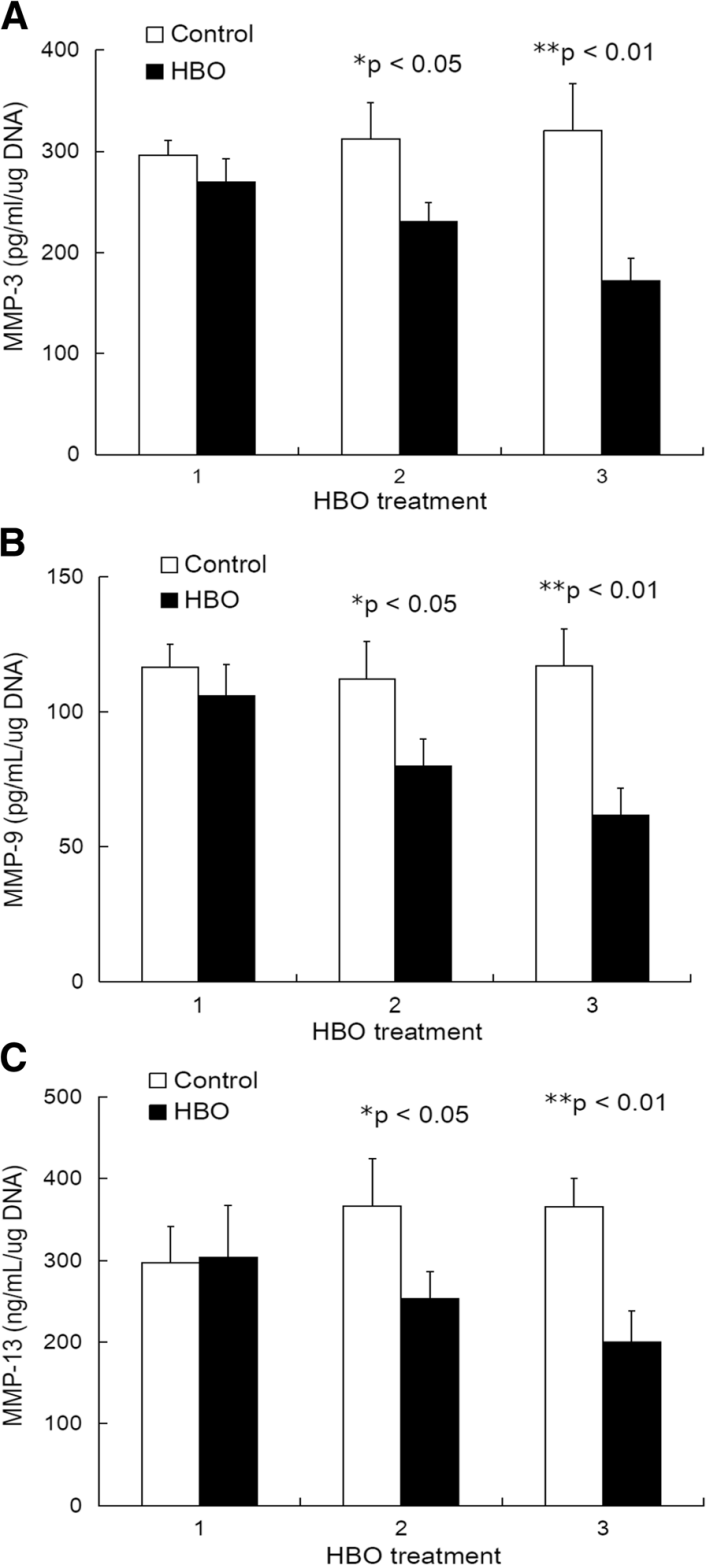
Effect of HBO on MMP-3, MMP-9, and MMP-13 secretion. **a** HBO treatment significantly inhibited MMP-3 secretion (*p* > 0.05, *p < 0.05, **p < 0.01; *n* = 4) by degenerated NPCs. **b** HBO treatment significantly inhibited MMP-9 secretion (*p* > 0.05, *p < 0.05, **p < 0.01; *n* = 4) by degenerated NPCs. **c** HBO treatment significantly inhibited MMP-13 secretion (*p* > 0.05, *p < 0.05, **p < 0.01; *n* = 4) by degenerated NPCs. The observed regulation of these MMPs could likely be a result of the decreased MAPK and NF-kB signaling following HBO treatment

Because MAPK signaling and NF-κB activation may contribute to IVD degradation by way of upregulated expression of MMPs, we performed ELISA analyses to assess the release of the pro-inflammatory cytokines MMP-3, MMP-9, and MMP-13 from HBO-treated NPCs (Fig. 8). Figure 8a shows the effect of three rounds of HBO treatment on MMP-3 secretion (HBO group vs. control group: 296.3 ± 14.6 vs. 269.7 ± 23.3, *p* > 0.05; 312.3 ± 35.4 vs. 231.0 ± 18.2, *p < 0.05; 320.4 ± 46.3 vs. 171.8 ± 22.8, **p < 0.01; *n* = 4). Figure 8b shows the effect of three rounds of HBO treatment on MMP-9 secretion (HBO group vs. control group: 116.6 ± 8.3 vs. 105.8 ± 11.8, *p* > 0.05; 112.1 ± 14.0 vs. 79.8 ± 10.0, *p < 0.05; 117.0 ± 13.8 vs. 61.6 ± 10.1, **p < 0.01; *n* = 4). Figure 8c shows the effect of three rounds of HBO treatment on MMP-13 secretion (HBO group vs. control group: 297.1 ± 44.4 vs. 304.1 ± 63.2, *p* > 0.05; 366.4 ± 58.1 vs. 253.1 ± 33.3, *p < 0.05; 365.3 ± 35.5 vs. 200.3 ± 37.9, **p < 0.01; *n* = 4). These results demonstrate that HBO treatment significantly inhibited MMP-3, MMP-9, and MMP-13 secretion from degenerated human NPCs.

## Discussion

MiR-107 is increasingly appreciated to serve key functions in humans [29, 30]. MiR-107 is significantly downregulated in inflamed colons [31]. Mir-107 expression was increased in the retina following hyperoxia [32]. The decreased expression of miR-107 in the chondrocyte was significant in initiation and progression of osteoarthritis (OA) [27, 28]. The similarities between IDD and OA prompted us to explore and subsequently reveal the potential roles of MiR-107 in IDD. In this study, MiR-107 is one of the first identified miRNAs upregulated in human degenerated NPCs after HBO treatment by microarray (Fig. 1a). We provide several lines of evidence here to demonstrate that miR-107 may be an important regulator in HMGB-1/RAGE inflammatory pathway in degenerated NPCs after HBO treatment.

First, we detected miR-107 expression in degenerated human NPCs after HBO treatment and confirmed this expression via real-time PCR (Fig. 1b). Hypoxia is a potent inducer of extracellular HMGB1 [33], and miR-107 can modulate the cellular response to hypoxia in tumors [34]. MiR-107 is downregulated during hypoxia [24], and HBO treatment increases the O_2_ levels in culture dishes to improve hypoxia conditions [8]. Our data indicated that HBO treatment increased miR-107 expression in NPCs (Fig. 1b), and HMGB1 was identified as a target gene of miR-107 (Figs. 2 and 3). These observations suggest that miR-107 might regulate inflammatory signaling via a mechanism targeting HMGB1, as HBO treatment significantly increased miR-107 expression and decreased HMGB1 production in degenerated NPCs (Figs. 1, 2, and 3).

Second, expression of HMGB1 [15] and RAGE [17, 18] was upregulated in degenerated discs. HMGB1 secretion is a known indicator of severe cellular damage and induces an inflammatory response in neighboring cells by interacting with receptors, such as RAGE on human OA chondrocytes [35] or bronchial epithelial cells (HBECs) [19]. HMGB1 is released from the cells after translocation from the nucleus to the cytoplasm or outside of the cell [36], indicating that secretion of HMGB1 may be associated with activation of the inflammasome complex [37]. In this study, HBO treatment was shown to significantly decrease the mRNA and protein expression levels of HMGB1 and RAGE in degenerated NPCs (Fig. 4). In addition, HMGB1 secretion from the cell was also downregulated after HBO treatment (Fig. 5). Inhibition of HMGB1-RAGE signaling, thus, appears to be a promising approach for limiting the inflammatory activity of HMGB1.

We next examined whether extracellular HMGB1 interacts with RAGE to activate the NF-κB or MAPK signal pathways after HBO treatment. Three major groups of distinctly regulated MAPK cascades in humans are known to lead to altered gene expression: extracellular signal-regulated kinase 1/2 (ERK1/2), c-Jun N-terminal kinase (JNK), and p38 MAP kinase. Activation (phosphorylation) of the MAPK pathway has been characterized by pathological changes in inflammatory or apoptotic processes [38], and treatment with HMGB1 results in phosphorylation of the ERK-1/2 MAPK and the p65 subunit of NF-κB in cultured chondrocytes [1], both of which are well-characterized mediators of RAGE signaling [11, 39]. Hypoxia induces MAPK activity in rat NPCs [12], and suppression of MAPK phosphorylation plays a key role in the protection of NPCs after HBO treatment [13]. In our previous study, we demonstrated that HBO suppresses MAPK signaling in degenerated human NPCs [8]. Consistent with these reports, the results in the present study suggested that the reduced HMGB1 secretion (Fig. 5) and RAGE expression (Fig. 4) after HBO treatment likely served to suppressed p38 MAPK, ERK, and JNK phosphorylation in NPCs (Fig. 6). As the inhibition of HMGB1 and RAGE by HBO treatment demonstrates an effective anti-inflammatory action, these results support the idea that HBO might play a role in remedying degeneration of NPCs via downregulation of HMGB1/RAGE/MAPK signaling.

In most cell types, NF-κB is composed of a p65/p50 heterodimer, and IκBα, IκBβ, IκBγ, and IκBε reside primarily in the cytoplasm and function to prevent NF-κB translocation into the nucleus. Under normal conditions, NF-κB is in an inactive state and mainly located in the cytoplasm. Once activated, the p65 subunit dissociates from its inhibitor, IκBα, and translocates from the cytoplasm to the nucleus to activate the transcription of its target genes [28]. Hypoxia induces inflammatory responses by activating NF-κB, which is the major mediator of inflammation and controls transcriptional programs that execute and regulate inflammatory responses [32]. HBO treatment reduces the inflammatory response in patients with acute pancreatitis by upregulating IκB activation and downregulating of NF-κB levels in granulocytes [29]. By investigating the HMGB1/RAGE/NF-κB signaling pathway in degenerated NPCs, we demonstrated that IκB expression was upregulated in response to HBO treatment and this decreased the translocation of NF-κB from the cytosol into the nucleus (Fig. 7). NF-κB–mediated iNOS mRNA and protein (Fig. 4) expression was subsequently downregulated after HBO intervention. These results suggest that HBO plays a role in countering the degeneration of NPCs by downregulating HMGB1/RAGE/NF-κB/iNOS signaling expression.

Three, recent studies suggest that expression of MMP-3, MMP-9, and MMP-13 is increased in degenerative disc disease [40, 41]. MMP-13 is a collagenase that preferentially cleaves Type II collagen relative to Types I and III [40], MMP-3 digests noncollagenous matrix proteins and denatured collagen molecules, and MMP-9 degrades denatured collagen molecules and basement membrane collagens [42]. Enzymes that mediate matrix degradation, including MMPs, are upregulated during the process of IVD degeneration and aging, resulting in increased matrix degradation [43]. Additionally, the increased expression and synthesis of MMPs and inflammatory factors is mediated by specific signal transduction events in IVDs involving the NF-κB– and MAPK-mediated pathways [5, 44]. HBO treatment significantly inhibits the secretion of MMP-3 [8], MMP-9 [45], and MMP-13 [46] in different cell types, and consistent with these findings, our data suggested that the phosphorylation of p38-MAPK, ERK-1/2, and JNK was suppressed in response to HBO treatment (Fig. 6), which may result in the downregulation of MMP-3 (Fig. 8a), MMP-9 (Fig. 8b), and MMP-13 (Fig. 8c) production. In addition, HBO increases the protein expression of IκBα, which leads to downregulation of NF-κB signaling (Fig. 7) and MMP-3 (Fig. 8a), MMP-9 (Fig. 8b), and MMP-13 secretion (Fig. 8c). As such, it is apparent that HBO treatments can reduce MMP-associated extracellular matrix damage and promote the repair of degenerated IVD.

Recently, Mir-107 could inhibit cell autophagy, proliferation, and migration of breast cancer cells by targeting HMGB1 has been reported [39]. In the present study, we showed that miR-107 was upregulated where HMGB1 is downregulated in IVD cells, indicating a reverse correlation between miR-107 and HMGB1. In addition, miR-107 was directly binding to the 3′ UTR region of HMGB1 and led to its degradation. Relative mRNA and protein expression of HMGB1 was significantly lower in presence of overexpressed miR-107 after HBO treatment.

Hypoxia can enhance the angiogenic ability of IVD during inflammatory reactions and cause progress in development of DDD via extracellular matrix regulation in vitro [47]. In the present study, HBO treatment increased oxygen tension and upregulated miR-107 expression in degenerated human IVD cells. Our results providing evidence that miRNA 107 can be used as a clinical therapeutic agent in degenerating disc for biological repair as the vascularity of the degenerating disc is increased.

Expression of Toll-like receptors (TLR) was detected in isolated human IVD cells [48]. TLR-ligand interaction can lead to activation of NF-κB and MAPK, which has been detected under specific conditions in IVD cells in vitro and in vivo [49, 50]. HBO treatment suppressed TLRs expression in degenerated IVD cells (unpublished data). The effects of HBO on HMGB1/TLR/NF-κB signaling in degenerative IVD disease are needed to be further investigation.

Many factors such as aging, spine deformities and diseases, spine injuries, and genetic factors are involved in the pathogenesis of IDD [51]. There is accumulating evidence that miRNAs are associated with the prognosis and progression of several diseases and may serve as future targets for gene therapy [52]. The role of miRNA in human degenerative disc disease is also beginning to be explored. Epigenetic silencing of miRNA-143 regulates apoptosis by targeting BCL2 in human intervertebral disc degeneration [53]. In addition, mir-98 expression level was significantly lower in degenerative NP tissues and significantly correlated with disc degeneration grade [54]. The miR-141 expression level in NP tissues from IDD patients was positively correlated with the disc degeneration grade [55].

When grown in monolayer culture, human disc cells assume a flattened, spindle-shaped morphology. The proteoglycans chondroitin sulfate and keratan sulfate showed abundant localizations between and around the cultured cells. Monolayer cultures showed only very sparse, very rare, small localizations for either type I or II collagen. However, three-dimensional culture results in a rounded cell phenotype, increased proteoglycan synthesis compared to monolayer grown cells, and formation of multi-celled colonies with ECM deposited around and between cells [56, 57].

HBO serves as primary or adjunctive therapy for a diverse range of medical conditions (ex. crush injury, traumatic ischemia, radiation necrosis of soft tissue, and bone) [58, 59]. One of the mechanisms is by increasing the oxygen diffusion to the tissues by raising dissolved oxygen level in plasma or body fluid. Because oxygen is a gas, the effectiveness of the HBO treatments is that the gases are pushed into the tissue not just delivered through the blood stream [58–60]. The basis of modulating pressure and oxygen concentration is to regulate cellular metabolism or tissue microenvironment. Therefore, the HBO treatment may be a good choice to study avascular tissue like IVD. In our previous animal studies, HBO treatment suppressed iNOS expression and apoptosis of cells in rabbit OA model [61] and rat degenerated IVD model [62]. HBO increased the oxygen tension in synovial fluid in rabbit OA model [61]. Recently, HBO therapy downregulated HMGB-1 and NF-κB expression in clinical patients with acute spinal cord injury also has been reported [63].

We note that our study has several limitations. In severe grades of discs degeneration (Pfirrmann Grading 4 o 5), it is difficult to demarcate NP from AF and the cells isolated are a mix and transformed population, affecting the results. HBO treatment also suppressed TLRs expression in degenerated IVD cells (unpublished data). The effects of HBO on HMGB1/TLR/NF-κB signaling in degenerative IVD disease are needed to be further investigation.

## Conclusions

The results of this study indicate that HBO treatment of degenerated NPCs exerts a protective effect by mitigating inflammation and its activation. These effects are induced by the inhibition of the miR-107/HMGB1/RAGE/MAPK and NF-κB signaling pathways and consequent suppression of pro-inflammatory cytokines and MMPs levels.

## Abbreviations

3′ UTR: 3′ untranslated region
CM: Conditioned media
ERK: Signal-regulated kinase
FADD: Fas-associated protein with death domain
HBO: Hyperbaric oxygen
HMGB1: High-mobility group box 1
IDD: Intervertebral disc degeneration
IL-1: Interleukin-1
iNOS: Nitric oxide synthase
JNK: c-Jun N-terminal kinase
MAPK: Mitogen-activated protein kinase
miRNAs: microRNAs
MMP: Metalloprotease
Mut: Mutant variant
NF-κB: Nuclear factor-kappa B
NO: Nitric oxide
NPCs: Nucleus pulposus cells
PCR: Polymerase chain reaction
PGE2: Prostaglandin E2
RAGE: Receptor for advanced glycation end-products
RT: Reverse transcription
TNF-a: Tumor necrosis factor-alpha
WT: Wild type
